# Coaxial nanofibers outperform uniaxial nanofibers for the loading and release of pyrroloquinoline quinone (PQQ) for biomedical applications

**DOI:** 10.1039/d0na00311e

**Published:** 2020-06-08

**Authors:** Sara Ibrahim, Marwan Y. Rezk, Mohammed Ismail, Taghrid Abdelrahman, Mona Sharkawy, Ahmed Abdellatif, Nageh K. Allam

**Affiliations:** Energy Materials Laboratory, School of Sciences and Engineering, The American University in Cairo New Cairo 11835 Egypt nageh.allam@aucegypt.edu; Zoology Department, Faculty of Science, Cairo University Giza 12613 Egypt; Biology Department, School of Sciences and Engineering, American University in Cairo New Cairo 11835 Egypt

## Abstract

Pyrroloquinoline quinone (PQQ), present in breast milk and various foods, is highly recommended as an antioxidant, anti-inflammatory agent, and a cofactor in redox reactions in several biomedical fields. Moreover, PQQ has neuroprotective effects on nervous system disorders and immunosuppressive effects on different diseases. Herein, we report on the optimum fabrication of electrospun CS/PVA coaxial, core/shell, and uniaxial nanofibers. The morphological, elemental, and chemical structure of the fabricated nanofibers were investigated and discussed. PQQ, as a drug, was loaded on the uniaxial nanofibers and in the core of the coaxial nanofibers and the sustained and controlled release of PQQ was compared and discussed. The results revealed the privilege of the coaxial over the uniaxial nanofibers in the sustained release and reduction of the initial burst of PQQ. Remarkably, the results revealed a higher degree of swelling for CS/PVA hollow nanofibers compared to that of the uniaxial and the coaxial nanofibers. The coaxial nanofibers showed a lower release rate than the uniaxial nanofibers. Moreover, the CS/PVA coaxial nanofibers loaded with PQQ were found to enhance cell viability and proliferation. Therefore, the CS/PVA coaxial nanofibers loaded with PQQ assembly is considered a superior drug delivery system for PQQ release.

## Abstract 2

Pyrroloquinoline quinone (PQQ), present in breast milk and various foods, is highly recommended as an antioxidant, anti-inflammatory agent, and a cofactor in redox reactions in several biomedical fields.

## Graphical abstract

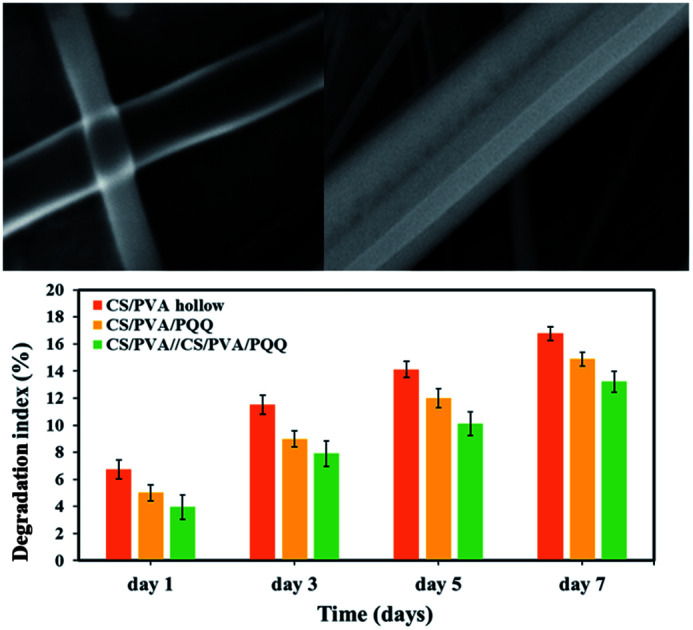

## Introduction

The process of forming nanofibers using an applied external field has been reported for the first time by Formhals in 1934.^1^ This process is known as electrospinning, a technique in which fibers are formed *via* charging polymer solutions electrically using high voltage and obtaining them at a specific feed rate. The obtained nanofibers, from the aforementioned technique, have diameters that range from a few nanometers to several micrometers.^2^ The applied external voltage causes repulsive forces within the polymer's solution to exceed the surface tension of a solution droplet at the tip of a needle. Consequently, it creates deformation upon the polymer solution's spherical droplet to elongate forming a Taylor cone, which in turn creates the strands of nanofibers. In fact, the created nanofibers still have to travel a sufficient distance from the needle to the collector to allow them to dry out from any remaining solvent, producing dry, well-separated solid nanofibers.^3^ There are numerous factors that can affect the fibers' morphology and diameter including the polymer viscosity, humidity, flow rate, tip-to-collector distance (TCD), and applied voltage.^4–7^ Moreover, the accumulation of high surface area and high porosity nanofibers to form a membrane is a perfect candidate to be used in a wide range of medical applications, including tissue engineering and drug delivery.^8^

Coaxial electrospinning is an improvement of the uniaxial electrospinning process in which two blunt-tip needles are connected to a high voltage with the same or different flow rates. Coaxial electrospinning provides nanofibers with a different and separate polymer solution for the core and shell, a tool used to fabricate hollow nanofibers or fibers with a varying core–shell.^9–11^ In comparison with uniaxial nanofibers, coaxial fibers have many advantages where the coaxial technique enables the spinning of the material, which is not easily spun *via* uniaxial electrospinning.^12,13^ Moreover, in medical applications, sensitive bioactive substances such as antibiotics, drugs, DNA, and proteins, which are spun as the core solution, can be safely isolated from the harsh environment by the shell layer of nanofibers. Subsequently, controlling the drug release is possible *via* tuning the thickness of the core and shell.^13,14^ In addition, similar work has been done using novel biomaterial coaxial nanofibers for bone and cartilage regeneration.^15,16^

In the present study, chitosan (CS) and polyvinyl alcohol (PVA) were selected as core and shell polymers, respectively. CS is a natural biopolymer with a repeated polysaccharide unit, which consists of *N*-acetyl glucosamine and glucosamine linked by glycosidic bonds.^17^ Many previous studies reported that the electrospinning of pure CS is not easy due to high viscosity, limited solubility and the strong molecular interaction of CS.^18,19^ Therefore, to facilitate the synthesis of CS nanofibers, chitosan/other polymer blends such as PVA have been used.^19,20^ CS/PVA nanofibers have been used in many biomedical applications such as tissue engineering, drug delivery and wound healing due to their antibacterial, biocompatible and biodegradable properties. Our study selects coaxial CS/PVA nanofibers loaded with the natural component pyrroloquinoline quinone (PQQ) as a promising material for biomedical applications such as a drug delivery system for many medical applications.

PQQ is an anionic, water-soluble compound that has been discovered in several fruits, vegetables, breast milk, and tissues of mammals.^21,22^ PQQ was initially identified as a redox enzyme cofactor and later proved to be an essential nutrient for animal growth. It has been reported that the removal of PQQ from the diet causes growth impairment in animals.^23,24^ As an essential nutrient, PQQ has been classified as a new B vitamin with many other beneficial effects, such as anti-inflammatory,^25^ hepatoprotective,^26^ cardioprotective,^27^ and antioxidative properties.^28,29^ Furthermore, much evidence shows that PQQ is a key player in the central and peripheral nervous systems. For example, PQQ shields the brain against reversible middle cerebral artery occlusion,^28^ and increases the stimulus-response and the nerve growth factor levels in astroglial cells.^30^ In addition, it enhances the locomotor function after spinal cord injury in rats. This is achieved by lessening the secondary neuronal damage following primary mechanical injury^31^ and it further improves the regeneration of rat sciatic nerves.^32^ Consequently, PQQ is considered a perfect candidate for many medical applications and a key solution for complications, especially in the nervous system.

Herein, we demonstrate the fabrication of CS/PVA uniaxial and coaxial nanofibers and compare their efficiency for the sustained release of pyrroloquinoline quinone (PQQ). The fabricated materials were fully characterized and compared. Also, the cell viability and proliferation of the cells on both classes of nanofibers were evaluated for use in biomedical applications.

## Materials & methods

### • Materials

CS (deacetylation degree: 82.6%, *M*_w_: 190.000–310.000 Da), PVA (*M*_w_ 89 000–98 000, 99+% hydrolyzed), and acetic acid (AA, 99+%) were purchased from Merck, USA. PQQ was purchased from Sigma (USA).

### • Preparation of nanofiber solutions

The prepared solutions of CS and PVA were used for uniaxial and coaxial electrospinning as previously reported by Ibrahim *et al.*^33^ Briefly:

(1) 3 g of CS (3% w/v) were dissolved in 100 ml of AA/distilled water solution (2% v/v) and heated in a water bath at 90 °C with vigorous stirring for 2 h until complete dissolution, and used as a stock.

(2) 20 g of PVA (20% w/v) were dissolved in 100 ml of distilled water and heated in a water bath at 90 °C with vigorous for 3 h until complete dissolution, and used as a stock.

(3) 70 ml of PVA/distilled water solution were mixed with 30 ml of CS/AA/distilled water solution in volume ratios of 30/70 of CS/PVA, with vigorous stirring till reaching a homogenous solution.^34^

(4) After complete homogenization, 40 ml of distilled water were added to 60 ml of CS/PVA homogenous mixture and were allowed to vigorously stir for 1 h at 60 °C to maintain the viscosity of the solution.^33,34^ The pH and viscosity of various prepared solutions were adjusted using a pH meter and a viscometer.

(5) For drug loading, PQQ (15% w/v) was added to the mixture, where PQQ solution was prepared by dissolving in distilled water. The CS/PVA solution loaded with PQQ was homogenized using a homogenizer for 1 h at 3000 rpm. Finally, the solutions (CS/PVA and CS/PVA loaded with PQQ) were left on the bench overnight to get rid of air bubbles before the electrospinning process.

### • Electrospinning process of solutions

An electrospinning apparatus (MECC Nanon-01A, Japan) was used to fabricate nanofibers with hollow and core/shell structures. The blunt-tip coaxial needle was composed of a 23 G (i.d. 0.33 mm, o.d. 0.63 mm) inner needle concentrically mounted on an 18 G (i.d. 0.96 mm, o.d. 1.26 mm) outer needle where each of them has a separate syringe pump. In order to fabricate uniaxial CS/PVA/PQQ nanofibers, CS/PVA/PQQ solution was loaded in the syringe of the uniaxial set with an applied voltage of 27 kV, a feed rate of 0.4 ml h^−1^ and a TCD of 15 cm. For the hollow CS/PVA nanofibers, CS/PVA solution was loaded in the syringe of the outer needle only while the inner needle was filled with air. The applied voltage, flow rate, and TCD were 27 kV, 0.4 ml h^−1^, and 15 cm, respectively. The temperature and humidity were controlled at 25 °C and 30–40%. However, for the fabrication of CS/PVA uniaxial nanofibers and CS/PVA/PQQ coaxial nanofibers, CS/PVA and CS/PVA/PQQ solutions were loaded in two separate syringes. The CS/PVA solution was loaded as the outer needle (shell) and the CS/PVA/PQQ solution was loaded in another syringe as the inner needle (core). The applied voltage and TCD were 27 kV and 15 cm, respectively. The temperature and humidity were controlled at 25 °C and 30–40%. The flow rate of the outer needle's syringe was 0.4 ml h^−1^, while the flow rate of the inner needle's syringe was 0.3 ml h^−1^.

### • Nanofiber testing

The elemental and morphological analysis of the fabricated nanofibers were performed using a field emission scanning microscope (FSEM, Zeiss SEM ultra 60) and a transmission electron microscope (TEM, JEM-1230, Jeol, Japan). To confirm the chemical bonding, Fourier Transform Infrared spectroscopy (FT-IR, Thermo Scientific Nicolet 8700, USA) was used to characterize the chemical structure of the uniaxial CS/PVA/PQQ, CS/PVA hollow and CS/PVA/PQQ coaxial nanofibers.

### • Degree of swelling and degradation experiments

CS/PVA uniaxial, hollow and core/shell membranes were cut into small pieces of 3 × 3 cm and the initial weight of nanofibers (*W*_i_) was recorded. For the swelling test, the pieces of nanofibers were immersed in phosphate buffer solution (PBS; pH 7.4) at 37 °C for 1, 3, and 7 days, and the samples were weighed again immediately after removing them from PBS and recorded as (*W*). Subsequently, the excess amount of PBS absorbed with filter paper and the weight of the nanofiber sample (*W*_x_) were recorded. The degree of swelling of the nanofibers was calculated using eqn (1):1
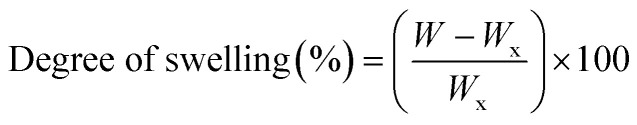
where *W* is the weight of swollen nanofiber samples by immersion in PBS, pH = 7.4 at 37 °C for 1, 2 and 3 days. *W*_d_ is the weight of the dried nanofiber sample.

Regarding the degradation test, the nanofibers were immersed in phosphate buffer solution (PBS; pH 7.4) at 37 °C for 1, 3, and 7 days, and the nanofibers were dried in a vacuum oven for 24 h and the weight of the dried nanofiber sample (*W*_d_) was recorded. The degradation index (%) was calculated using eqn (2):2
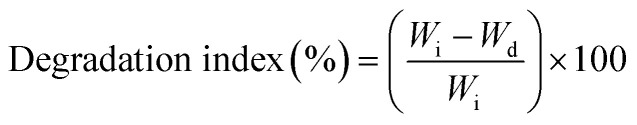
where *W*_i_ is the initial weight of the nanofibers.

### • PQQ release experiment (*in vitro*)

Using a Franz diffusion cell apparatus (Automated Microette PlusTM; Hanson Research, USA) CS/PVA/PQQ uniaxial nanofibers, and CS/PVA and CS/PVA/PQQ coaxial nanofibers were prepared to test drug release. The area of Franz diffusion cells was 1.76 cm^2^ with a receptor compartment volume of 6.8 ml. The test was performed using a cellulose membrane with a molecular weight cut-off value of 1400 g mol^−1^ (Sigma Aldrich, USA). The nanofibers were placed in the donor compartment, while PBS with pH 7.4 was placed in the receptor compartment with adjusting temperature at 37 °C and the stirring rate at 200 rpm. Samples (2 ml) were taken at 24, 72, 120 and 168 hours and diluted to measure the PQQ concentration by using a UV-visible spectrophotometer at a *λ*_max_ of 280 nm.

### • Antibacterial activity test

The antimicrobial activity of the nanofiber samples was determined using a modified Kirby–Bauer disc diffusion method.^35^ Briefly, 100 μl of the test bacteria were grown in 10 ml of fresh media until they reached a count of approximately 108 cells per ml for bacteria.^36^ 100 μl of microbial suspension was spread onto agar plates corresponding to the broth in which they were maintained. Isolated colonies of each organism that might be playing a pathogenic role should be selected from primary agar plates and tested for susceptibility by the disc diffusion method.^37,38^ Gram(+) bacteria such as *Staphylococcus aureus* and *Bacillus subtilis* and Gram(−) bacteria such as *Escherichia coli* and *Pseudomonas aeruginosa* were incubated at 35–37 °C for 24–48. The diameters of the inhibition zones were measured in millimeters.^35^ Standard discs of ampicillin (antibacterial agent) served as positive controls for antimicrobial activity but filter discs impregnated with 10 μl of solvent (distilled water, chloroform, DMSO) were used as a negative control. Blank paper disks (Schleicher & Schuell, Spain) with a diameter of 8.0 mm were impregnated with 10 μl of tested concentration of the stock solutions. When a filter paper disc saturated with a tested chemical is placed on agar the chemical will diffuse from the disc into the agar. This diffusion will place the chemical in the agar only around the disc. The size of the area of chemical infiltration around the disc is determined *via* the solubility of the chemical and its molecular size. If an organism is placed on the agar it will not grow in the area around the disc if it is susceptible to the chemical. This area of no growth around the disc is known as a “Zone of inhibition” or “Clear zone”. For the disc diffusion, the calibers of the National Committee for Clinical Laboratory Standards^38^ were used to quantitatively measure the inhibition zone diameters. The agar-based methods including tests such as Etest and disk diffusion can be good substitutes for broth-based methods due to their simplicity, higher accuracy, and the less time taken for the test.^39,40^

### • MTT cytotoxicity assessment

Regarding the evaluation of human fibroblast cell proliferation (*in vitro*), in a Fibrolife® serum-free medium, human dermal fibroblasts, adult (HDFa) (American Type Culture Collection® PCS-201-012™, USA) cell cultures were cultivated at 37 °C and 5% CO_2_ in T75 culture flasks (Corning®, USA) for 24–27 hours. In order to determine the effect of CS/PVA hollow and CS/PVA/PQQ core/shell nanofibers on the proliferation of HDFa *via* 3-(4,5-dimethyl-2-thiazolyl)-2,5-diphenyl-2*H*-tetrazolium bromide (MTT) assay the percentage viable cell was used^41^ as follows:

(1) CS/PVA uniaxial, hollow and core/shell nanofibers were suspended (three replicates for each sample) with HDFa in a Fibrolife® serum free medium at a concentration of 5 × 10^4^ cell per well in Corning® 96-well tissue culture plates.

(2) Then the plates were incubated for 72 hours.

(3) Vehicle control with DMSO was run for each 96 well plate (untreated cells).

(4) After incubating for 72 h, the number of viable cells was then determined by adding 10 μl of 12 mM MTT stock solution (5 mg of MTT in 1 ml of phosphate buffer saline pH 7.4) into each well, including also the untreated control.^42,43^

(5) The 96-well plates were then incubated at 37 °C and 5% CO_2_ for 4 hours.

(6) The cells were periodically viewed under an inverted microscope (Olympus BX63 Life Science, Japan) to detect the presence of intracellular punctuate purple precipitation. The optical density was measured at 490 nm with a microplate reader (680 XR reader, BIORAD, Hercules, CA, USA) to determine the number of viable cells.

(7) The percentage of viability was calculated using the following eqn (3):3
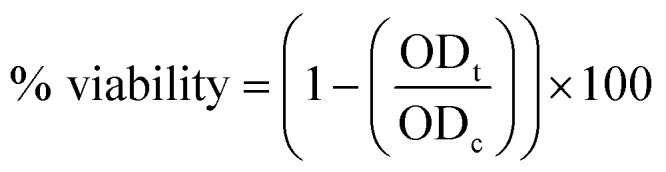
where OD_t_ is the mean optical density of the wells treated with the tested samples (drug or the selected formulation), while OD_c_ is the mean optical density of the untreated cells.

## Results & discussion

### • Morphological, structural, and elemental analysis

**Fig. 1:**
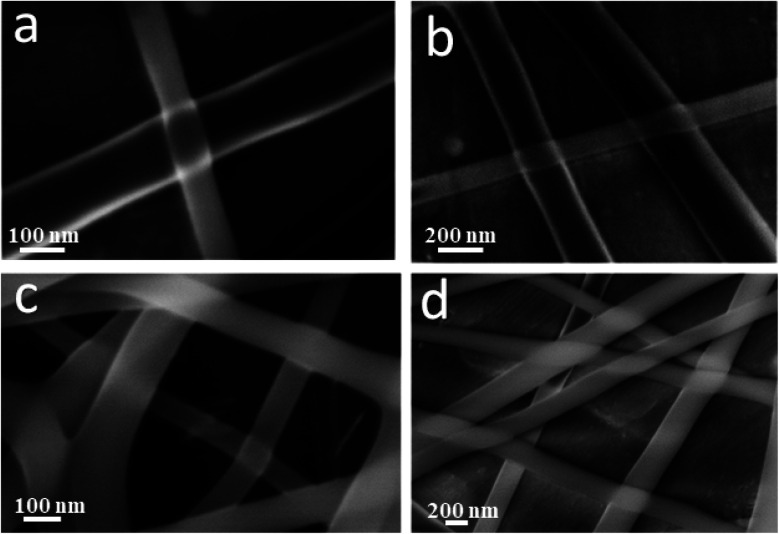
FESEM images of the as-synthesized hollow CS/PVA nanofibers.

The coaxial electrospinning technique was used to fabricate CS/PVA hollow nanofibers using acetic acid/distilled water solution as a solvent. On one hand, CS/PVA nanofibers, as a shell, were synthesized under constant processing conditions where the concentration of CS/PVA mixtures was 50%, with a voltage of 27 kV, TCD of 15 cm, humidity 30–40%, and flow rate of 0.4 ml h^−1^. On the other hand, the core was made hollow by filling the syringe with air using the same flow rate of the shell solution (0.4 ml h^−1^). Fig. 1 shows the as-synthesized hollow nanofibers prepared under the aforementioned conditions, revealing the good uniformity with a fine structure that is free from beads. Note the transparency of the nanofibers, which indicates the hollow core of the as-synthesized nanofibers.

**Fig. 2:**
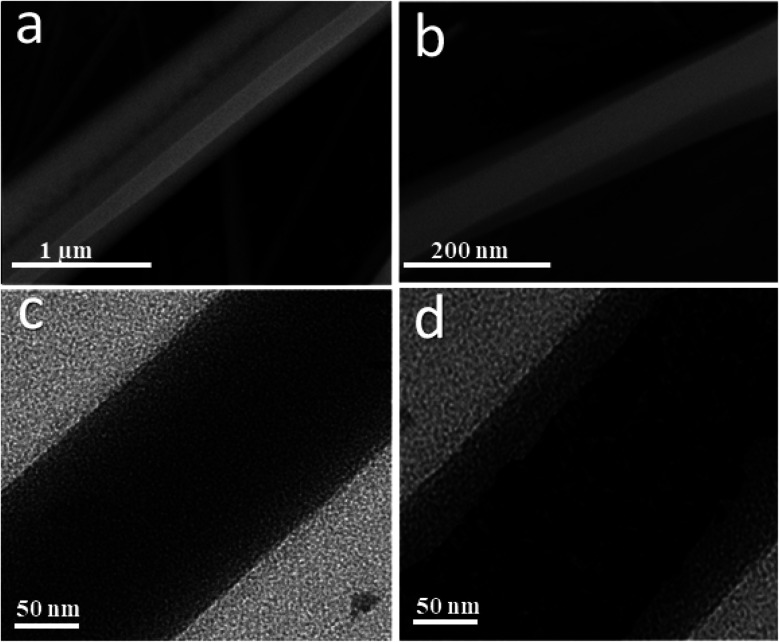
(a and b) FSEM of coaxial CS/PVA//CS/PVA/PQQ (shell//core), and TEM images of the (c) hollow and (d) coaxial nanofibers.

Fig. 2a,b show the FESEM images of the coaxial CS/PVA//CS/PVA/PQQ (shell//core) nanofibers prepared under constant processing conditions (27 kV, TCD 15 cm, humidity 30–40% and flow rate of 0.3 ml h^−1^ for core solution and 0.4 ml h^−1^ for shell solution). Note that the coaxial nanofibers exhibited uniform fibers with a shell/core structure. The presence of core fibers with a smaller diameter within the shell fiber is evident. To further investigate the presence of the core/shell structure within the nanofibers, the core/shell as well as the hollow nanofibers were scanned by TEM. The TEM images shown in Fig. 2c and d depict the hollow and core/shell structure, respectively.

**Fig. 3:**
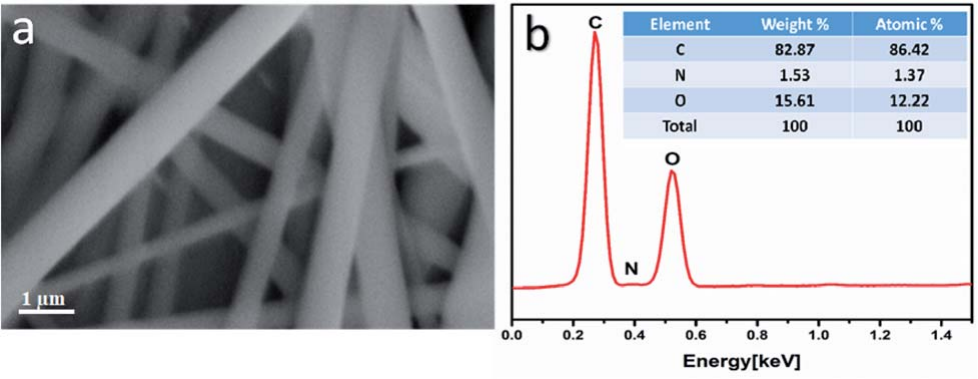
(a) FESEM images and (b) EDX elemental analysis of the uniaxial CS/PVA/PQQ nanofibers.

The morphology of the uniaxial CS/PVA/PQQ electrospun nanofibers was observed by FESEM. Fig. 3a reveals that the fabricated fibers have a smooth surface, uniform and completely bead-free. The elemental analysis of CS/PVA/PQQ is shown in Fig. 3b, indicating the existing elements as tabulated in the inset of Fig. 3b. The differences in the elemental peak intensities correspond to the initial elemental concentration used for the synthesis. It is quite lucid that carbon exhibits the most intense peak due to the high concentration of polymer used. The carbon constitutes the main backbone of all the polymer chains in the CS/PVA polymer. In addition, the remaining elements' (oxygen and nitrogen) peak intensities are directly proportionally to their atomic weight in the compound.

**Fig. 4:**
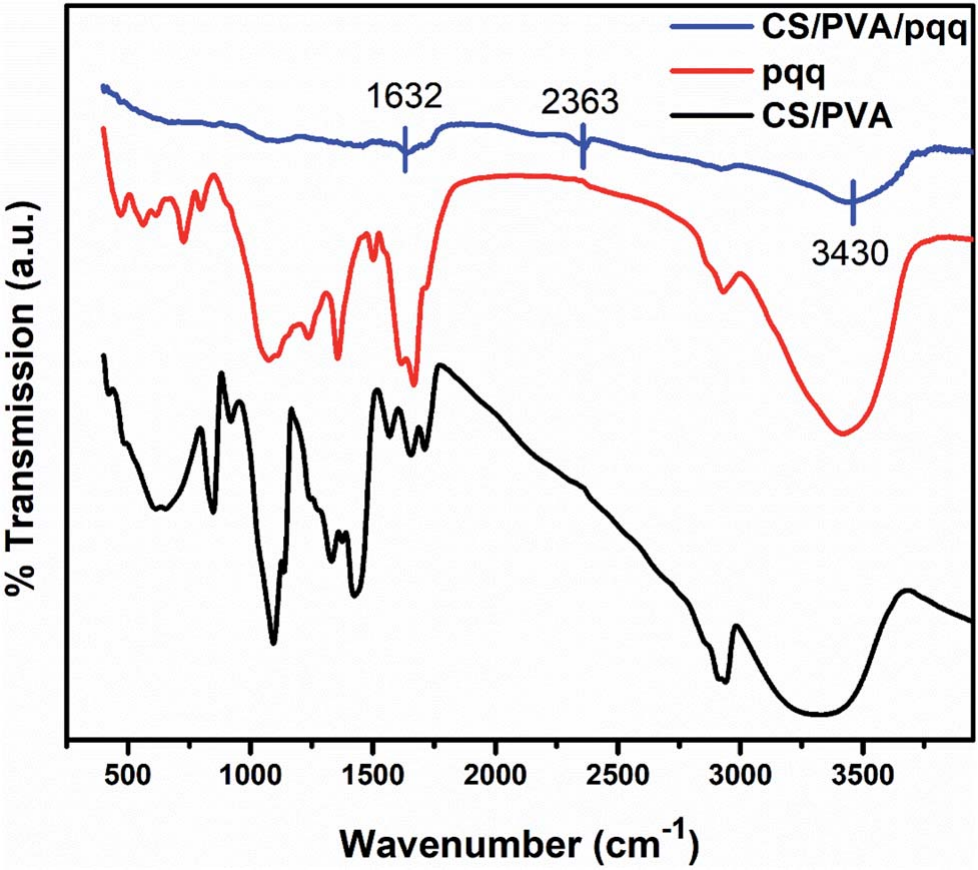
FTIR spectra of CS/PVA, CS/PVA/PQQ, and PQQ.

The FTIR spectra were collected to elucidate the chemical bonding for CS/PVA, PQQ, and CS/PVA/PQQ as shown in Fig. 4. The spectra of CS/PVA show a series of characteristic peaks for chitosan and PVA. The peak at 844 cm^−1^ confirms the presence of a saccharide structure as it is a major characteristic peak of the C–H bending in chitosan. Moreover, the 1096 cm^−1^ peak can be attributed to the C–O stretching vibrational mode in chitosan. Also, the peaks at 1375 and 1415 cm^−1^ are characteristic of the CH_3_ symmetrical deformation mode. The peak at 1640 cm^−1^ can be ascribed to the amide bond in chitosan. The vibrational band between 2790 and 2980 cm^−1^ can be ascribed to the C–H stretching of the alkyl groups. Finally, the O–H stretching shown in the broad band from 2980–3660 cm^−1^ is caused by the intramolecular and intermolecular hydrogen bonding in PVA. The PQQ spectra show 4 peaks located between 1505 and 1723 cm^−1^ that are characteristic of the C=O stretching vibrations of the bonding between quinone and carboxyl groups. As is lucid from the CS/PVA/PQQ spectra, the reaction between PQQ and CS/PVA caused the minimization/loss of most of the peaks except for the minor two peaks located at 1632 and 2363 cm^−1^ and the minor broad band at 3430 cm^−1^. They were indicated on the figure due to their low transmission.

### • Degree of swelling and degradation

**Fig. 5:**
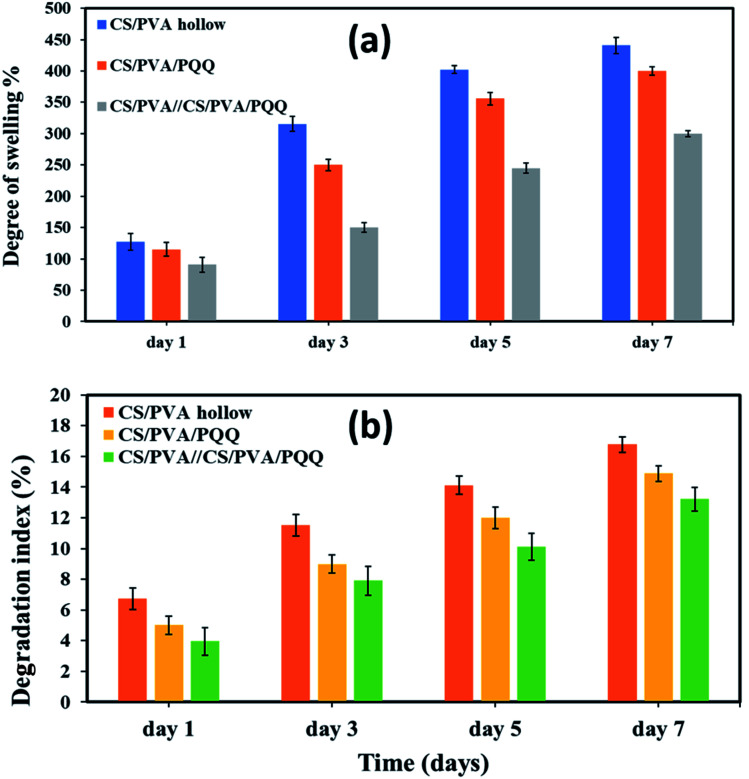
(a) Degree of swelling and (b) degradation index for the nanofibers on the 1st, 3rd, 5th, and 7th day.

To assess the physical properties of the fabricated nanofibers for biomedical applications, the degree of swelling and degradation are considered critical parameters that need to be quantitatively evaluated. The results of the degree of swelling are represented in Fig. 5a for the fabricated hollow, uniaxial, and coaxial nanofibers. The swelling volume of the CS/PVA hollow nanofiber was ∼127.23% for the first day and increased to reach 345.49% after three days. On the fifth day, the swelling degree reached 402.21% and then minimally increased to reach 440.77% on the seventh day. On the other hand, for the uniaxial CS/PVA/PQQ, the swelling volume was ∼115.23, 250.11, 355.79, and 400.10% for days one, three, five and seven, respectively, whereas, the swelling volume of CS/PVA//CS/PVA//PQQ coaxial nanofibers was ∼90.61% on the first day, 150.22% after three days, 244.96% for day five, and reaches 300.09% for day seven. Remarkably, the results revealed a higher degree of swelling for CS/PVA hollow nanofibers compared to that of the uniaxial and the coaxial nanofibers. For the coaxial nanofibers, the presence of CS/PVA/PQQ in the core decreased the swelling ratio of the coaxial nanofibers on the seventh day of the test from 440.77% for CS/PVA hollow and 400.10% for CS/PVA/PQQ uniaxial nanofibers to 300.09%. Furthermore, this reduction in the degree of swelling of the nanofibers can be ascribed to the presence of a core structure within the shell, resulting in increasing the rigidity of the nanofibers.

Fig. 5b shows the degree of degradation of the as-synthesized nanofibers. The slower the rate of degradation, the slower the release of PQQ, which is the most favorable characteristic in medical applications and sustained drug release systems. The results showed that the degradation index of CS/PVA hollow nanofibers is around 6.74% for day one, 11.52% for day three, 14.13% for day five, and 16.77% for day seven. For uniaxial CS/PVA/PQQ nanofibers, the degradation index recorded was 5.01, 8.99, 11.99, and 14.88% for the 1st, 3rd, 5th, and 7th day, respectively. However, the degradation rate of PVA/CS//CS/PVA/PQQ nanofibers is around 3.907% for day one, 7.90% for day three, 10.12% for day five, and 13.22% for day seven. Interestingly, it was clearly observed that the degradation rate for CS/PVA//CS/PVA/PQQ coaxial scaffolds was much slower than that of uniaxial and hollow nanofibers. The presence of CS/PVA in the core of coaxial nanofibers was helpful to reduce the degradation rate of fibers, because it takes more time to degrade this core part in comparison with the time consumed to degrade the uniaxial and hollow nanofibers.

### • *In vitro* PQQ release

**Fig. 6:**
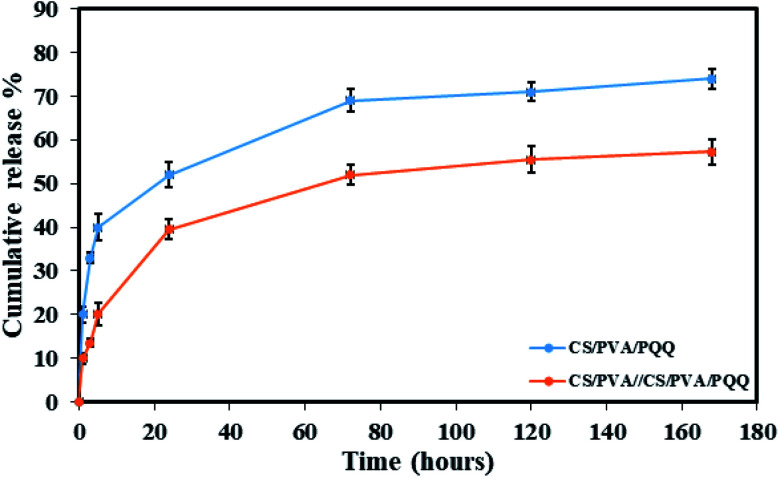
Release profiles of the CS/PVA/PQQ uniaxial nanofibers and CS/PVA//CS/PVA/PQQ coaxial nanofibers.

In order to verify the previous results and better compare the uniaxial and coaxial nanofibers, a release study of the PQQ was carried out by loading PQQ in the core of the CS/PVA//CS/PVA coaxial nanofibers as well as by loading the surface of CS/PVA uniaxial nanofibers, see Fig. 6. The concentration of the loaded PQQ in uniaxial and coaxial nanofibers for the drug release study was kept constant equivalent to 3.5 mg ml^−1^.^44^ During the first hour of the test, 20% of PQQ from the uniaxial nanofibers was released. However, only 10% of PQQ was released from the coaxial nanofibers without initial burst release. Moreover, the PQQ release from the core of the coaxial nanofibers reached 57.2% after 168 hours of the experiment. In contrast, in the same time interval of the test, a cumulative release of 74% of PQQ occurred from the uniaxial nanofibers. Note that the coaxial nanofibers have a lower release rate than the uniaxial nanofibers. Since PQQ exists in the core of coaxial nanofibers, the CS/PVA shell acted as a barrier to slow down the rate of release of PQQ. In comparison, the uniaxial nanofibers, in which PQQ was loaded on their surface, have shown a rapid release. The difference in the release profile shown in Fig. 6 is due to the presence of the CS/PVA shell layer. Normally, this shell layer acts as a barrier to mass transfer and thus reduces the release rate of PQQ from the coaxial nanofibers in comparison with the uniaxial nanofibers.^44^

### • Antibacterial test

**Fig. 7:**
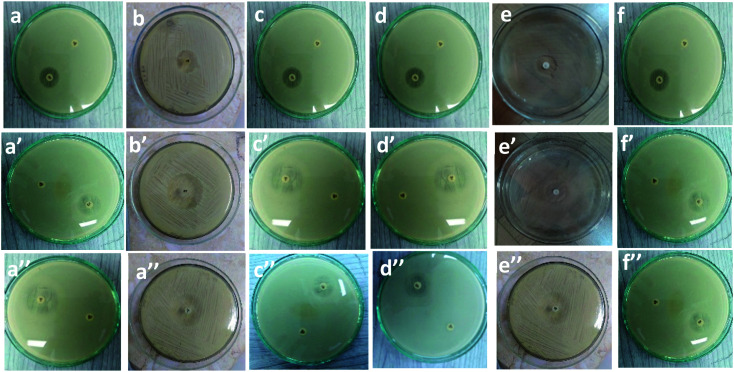
Inhibitory effect of (a–f): CS/PVA hollow, (a′–f′), uniaxial CS/PVA/PQQ and (a′′–f′′) coaxial CS/PVA//CS/PVA/PQQ nanofibers against different types of bacteria.

Table 1 and Fig. 7 show the inhibitory activity of CS/PVA hollow, CS/PVA/PQQ uniaxial and CS/PVA//CS/PVA/PQQ coaxial nanofibers in comparison with the antibacterial agent ampicillin. Generally, all the fabricated nanofibers inhibited the growth of the tested bacteria as indicated by the inhibition zone. The data in Table 1 show that CS/PVA hollow nanofibers showed the least inhibition of the growth of all tested bacteria. However, it still indicates a higher inhibition effect after loading PQQ on coaxial and uniaxial nanofibers. The results suggest that PQQ loaded on CS/PVA nanofibers as uniaxial or coaxial nanofibers can be used as a potential antibacterial scaffold for medical applications. Two main modes of action contribute to the resultant antibacterial effect indicated by the inhibition zones tabulated in Table 1. Firstly, chitosan can bind to the bacterial cell wall due to its negative charge. Subsequently, this binding can cause cell disruption and attachment to the DNA, which prevents DNA replication and cell death.^45^ Secondly, PQQ is naturally used in bacteria to change glucose into gluconic acid, which has a pH of 5–5.5.^46^ Since PQQ is added in excess to the bacteria (loaded on the nanofibers), more glucose is changed into gluconic acid, which consumes high energy. The bacteria used in the test work were at neutral to slightly alkaline pH. Thus, the accumulation of gluconic acid causes slight impairment of the bacterial behavior, motility, and growth.^47^ The aforementioned concluded modes of action can be confirmed by the less inhibition zone diameter of the hollow CS/PVA nanofiber due to the absence of PQQ. There is not much difference between the inhibition of Gram positive and Gram negative bacteria, which could be ascribed to the broad spectrum of the investigated coaxial/uniaxial nanofibers. In addition, it could also be attributed to the antibacterial mechanism that does not interact with the cell membrane.

**Table 1:** Antimicrobial tests against Gram^+^ and Gram^−^ bacteria^a^

Sample		Inhibition zone diameter (mm per mg sample)					
		Bacterial species					
		G^+^			G^−^		
		*Bacillus subtilis*	*Staphylococcus aureus*	*Streptococcus faecalis*	*Escherichia coli*	*Neisseria gonorrhoeae*	*Pseudomonas aeruginosa*
Standard	Ampicillin antibacterial agent	26 ± 0.1	21 ± 0.1	27 ± 0.3	25 ± 0.1	28 ± 0.4	26 ± 0.2
Control: DMSO		0 ± 0.0	0 ± 0.0	0 ± 0.0	0 ± 0.0	0 ± 0.0	0 ± 0.0
CS/PVA hollow		(a) 7 ± 0.3	(b) 7 ± 0.2	(c) 6 ± 0.4	(d) 6 ± 0.2	(e) 8 ± 0.2	(f) 6 ± 0.1
CS/PVA/PQQ uniaxial		(a′) 9 ± 0.2	(b′) 10 ± 0.3	(c′) 9 ± 0.1	(d′) 10 ± 0.5	(e′) 9 ± 0.3	(f′) 8 ± 0.1
CS/PVA//CS/PVA/PQQ		(a′′) 9 ± 0.2	(b′′) 9 ± 0.4	(c′′) 9 ± 0.3	(d′′) 9 ± 0.4	(e′′) 10 ± 0.3	(f′′) 8 ± 0.4

a Results were expressed as mean values of three independent samples ± standard deviations.

### • MTT cytotoxicity assessment

**Fig. 8:**
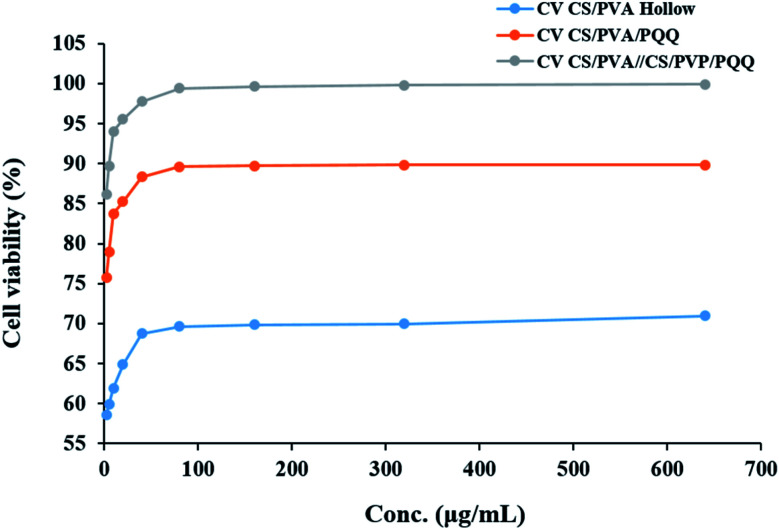
The effect of CS/PVA hollow, CS/PVA/PQQ uniaxial and CS/PVA//CS/PVA/PQQ coaxial nanofibers on the proliferation of HDFa cells determined by MTT assay.

In order to evaluate whether the loading of PQQ on CS/PVA nanofibers will be effective for many medical applications, the effect of CS/PVA hollow and PQQ loaded on CS/PVA nanofibers on proliferation of HDFa cells was examined using MTT assay, Fig. 8. The CS/PVA/PQQ nanofibers showed a significant increase in the proliferation of HDFa cells after 72 h of incubation, followed by CS/PVA hollow nanofibers (*p* < 0.05) (Fig. 8). The difference in the cell growth rate and cell numbers between CS/PVA and CS/PVA/PQQ nanofibers proves the role of PQQ loaded on CS/PVA nanofibers in increasing the proliferation of HDFa cells. Additionally, the comparison between uniaxial and coaxial nanofibers on the proliferation of HDFa cells reveals that coaxial nanofibers showed an increase in cell growth more than the uniaxial nanofibers, which could be attributed to low cytotoxicity. In addition, other work including Lu *et al.* has reported that the structure of the coaxial fibers resembles the extracellular matrix, which enhances cell proliferation and growth.^48^ Furthermore, the MTT assay results showed that the nanofiber scaffold had no cytotoxicity to HDFa and did not cause inhibition of proliferation or differentiation of HDFa.

## Conclusion

In conclusion, electrospun CS/PVA coaxial, core/shell, and uniaxial nanofibers were successfully fabricated. FESEM imaging revealed the good uniformity of the fabricated nanofibers with a fine structure that is free from beads. Moreover, their TEM images proved the hollow and core/shell structures. The elemental composition and the chemical bonding for CS/PVA, PQQ, and CS/PVA/PQQ were elucidated using EDX and FTIR analyses. The results proved the privilege of CS/PVA coaxial nanofibers in comparison with uniaxial nanofibers when loaded with PQQ for biomedical applications. The release study of PQQ showed that CS/PVA coaxial nanofibers have a lower release rate than uniaxial nanofibers with less initial release burst in comparison with uniaxial nanofibers. In addition, coaxial nanofibers have lower degradation and swelling rates than uniaxial nanofibers. Furthermore, it enhances the proliferation of HDFa cells. The obtained results strongly suggest the fabricated CS/PVA coaxial nanofibers loaded with PQQ in the core for use in many applications as an alternative to many complicated and expensive drug systems.

## Conflicts of interest

There are no conflicts to declare.

## Supplementary Material
